# Lysosomal solute and water transport

**DOI:** 10.1083/jcb.202109133

**Published:** 2022-10-11

**Authors:** Meiqin Hu, Nan Zhou, Weijie Cai, Haoxing Xu

**Affiliations:** 1 Collaborative Innovation Center of Yangtze River Delta Region Green Pharmaceuticals, College of Pharmaceutical Sciences, Zhejiang University of Technology, Hangzhou, China; 2 Department of Molecular, Cellular, and Developmental Biology, University of Michigan, Ann Arbor, MI; 3 Liangzhu Laboratory & Zhejiang University Medical Center, Hangzhou, China; 4 Department of Neurology, Second Affiliated Hospital of Zhejiang University Medical School, Hangzhou, China

## Abstract

Lysosomes mediate hydrolase-catalyzed macromolecule degradation to produce building block catabolites for reuse. Lysosome function requires an osmo-sensing machinery that regulates osmolytes (ions and organic solutes) and water flux. During hypoosmotic stress or when undigested materials accumulate, lysosomes become swollen and hypo-functional. As a membranous organelle filled with cargo macromolecules, catabolites, ions, and hydrolases, the lysosome must have mechanisms that regulate its shape and size while coordinating content exchange. In this review, we discussed the mechanisms that regulate lysosomal fusion and fission as well as swelling and condensation, with a focus on solute and water transport mechanisms across lysosomal membranes. Lysosomal H^+^, Na^+^, K^+^, Ca^2+^, and Cl^−^ channels and transporters sense trafficking and osmotic cues to regulate both solute flux and membrane trafficking. We also provide perspectives on how lysosomes may adjust the volume of themselves, the cytosol, and the cytoplasm through the control of lysosomal solute and water transport.

## Teaser

Hu et al. review the regulation of lysosome size by ion and water transport across lysosomal membranes.

## Introduction

Cells must maintain the volume constancy of the cytosol and cytoplasm (see text box) during osmotic water shifts, cell proliferation and death, and metabolically produced content changes (Hoffmann et al., 2009; Jentsch, 2016; Lang et al., 1998). Upon net water influx under hypotonic conditions, cells become swollen while the plasma membrane is stretched; if the membrane tension is not relieved, cells may undergo necrosis or burst (Ritter et al., 2021; Shubin et al., 2016). To protect cells from necrotic cell death, a regulatory volume decrease (RVD) response (see text box) must be triggered (Hoffmann et al., 2009). The RVD is believed to be mediated by plasma membrane Cl^−^/K^+^ efflux and subsequently passive extrusion of osmotically obligated H_2_O from the cytosol (Lang et al., 1998). In addition, exocytosis of intracellular vesicles may also help relieve plasma membrane tension (Okada et al., 1992; Truschel et al., 2002). Conversely, when cells are under hypertonic conditions or apoptotic cell death, a regulatory volume increase (RVI) response (see text box) is adaptively triggered, in which solute and water influx are involved (Hoffmann et al., 2009; Jentsch, 2016). Additionally, endocytosis of the extracellular fluid may also be involved in this response (Wang et al., 2011). Hence, the plasma membrane possesses a variety of mechanisms that regulate endocytosis and exocytosis, membrane stretching and relaxation, as well as solute and water flux.

Terminology

**Cytosolic volume**

The volume of the cytosol, which is the collection of the intracellular fluids of the cytoplasm besides intracellular membrane compartment, i.e., organelles.

**Cytoplasmic volume**

The volume of the cytoplasm, which contains all the components of the cell besides the plasma membrane, including the intracellular membranous organelles and the cytosol.

**Water potential**

A measure of the concentration of free water molecules, which is negatively proportional to the osmolarity of a solution. Water always moves from a system with a higher water potential to a system with a lower water potential.

**Osmotic pressure**

A pressure that is dependent on the concentrations of all the osmolytes in a solution that is required to prevent osmotic flow caused by water potential across a membrane. Water flows from a system with lower osmolarity to ta system with higher osmolarity.

**RVD/RVI**

Regulatory volume decrease/increase. The cell volume is usually referred to as the cytoplasmic volume. Cytosolic RVD/I is specifically referred to as regulatory cytosolic volume decrease/increase.

**Lysosomal storage diseases (LSDs)**

LSDs, which are disorders caused by toxic accumulations of undigested materials in the lysosome due to mutations in single genes.

Similar membrane addition/retrieval and solute/water flux mechanisms operate in intracellular membranous compartments, such as macropinosomes, phagosomes, autophagosomes, endosomes, and lysosomes (Freeman et al., 2020; Li et al., 2020). Primary lysosomes receive cargo through membrane fusion with autophagosomes and late endosomes (Huotari and Helenius, 2011; Luzio et al., 2007b). Hydrolase-mediated degradation then takes place in the resulting secondary lysosomes, such as autolysosomes, phagolysosomes, and endolysosomes (Ballabio and Bonifacino, 2020; Huotari and Helenius, 2011). Because the total volume is increased after membrane fusion due to a change in the surface-area-to-volume ratio, solute/water flux is involved in keeping the secondary lysosomes isotonic (Li et al., 2020). Upon completion of degradation, autolysosomes and endolysosome hybrids undergo membrane remodeling and fission: budding, vesiculation, tubulation, and scission (Luzio et al., 2007b; Saffi and Botelho, 2019). With additional membrane and content sorting, lysosomes are reformed or regenerated (Saffi and Botelho, 2019; Yang and Wang, 2021). To accommodate changes in the surface-area-to-volume ratio, volume resolution through solute/water extrusion must be executed prior to membrane fission, so that membrane deformation is permitted (Freeman et al., 2020; Luzio et al., 2007b; Pryor et al., 2000; Saric and Freeman, 2020; Zeziulia et al., 2022). Thus, lysosomal membrane tension undergoes constant changes during lysosomal membrane remodeling, trafficking, and sorting. However, the mechanisms that regulate solute and water flux in these processes are just beginning to be revealed (Freeman et al., 2020; Li et al., 2020; Saric and Freeman, 2020). An open question in the field is how the regulation of lysosomal membrane (surface area) and volume is coordinated (Li et al., 2020; Saric and Freeman, 2020). It is conceivable that certain cellular signals, acting as trafficking and osmotic cues, orchestrate both membrane fusion/fission and solute/water flux. In this review, we discuss how the osmolality (osmotic pressure) and membrane tension/curvature are sensed by lysosomal membrane proteins to control lysosome size/volume.

### Osmo-sensing is an integral part of lysosomal degradation

**Figure 1.**
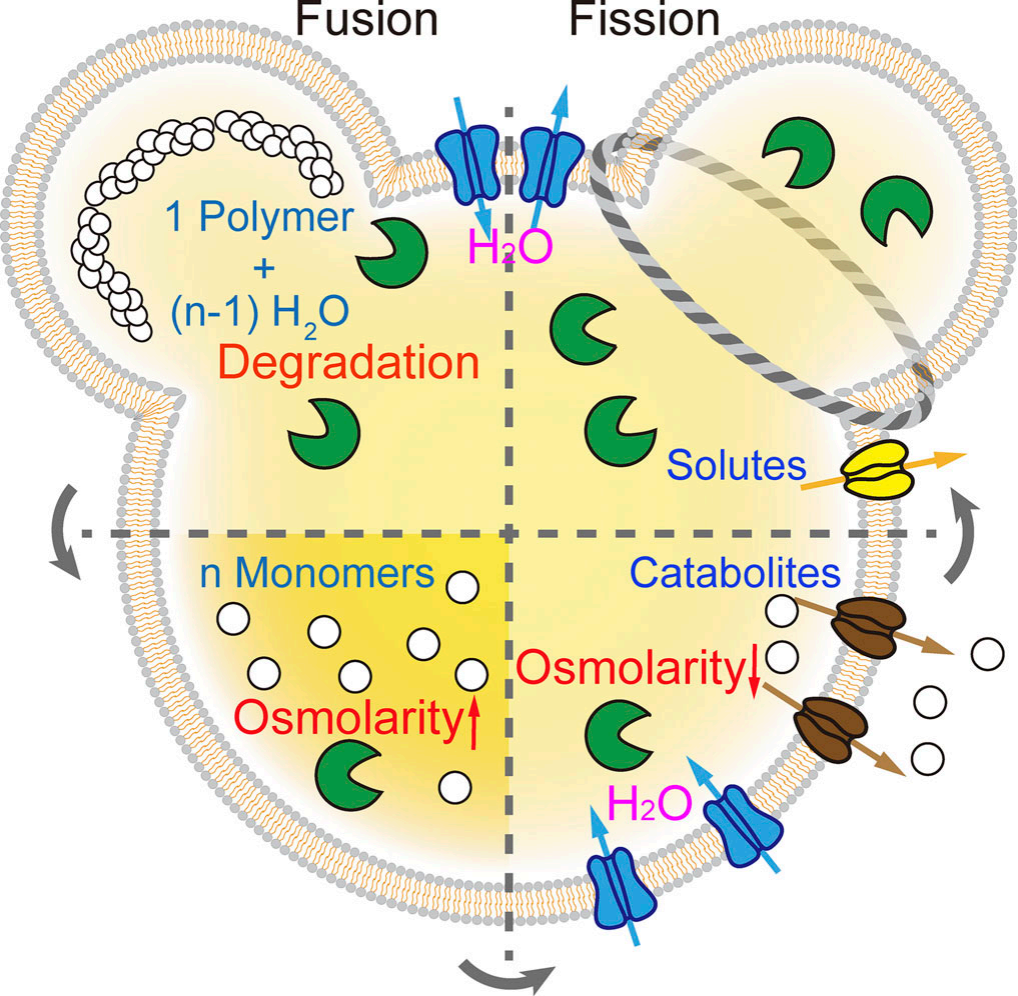
**Osmolarity changes in the lysosome lumen.** Cargo macromolecules (white chains) are delivered to the lysosomes through endosome-lysosome and autophagosome-lysosome membrane fusion. Upon lysosomal degradation mediated by hydrolases (green), macromolecules are digested into many molecules of catabolites (white circles), and the process consumes many molecules of H_2_O. Subsequently, there is a transient increase in luminal osmolality and a transient decrease in luminal water potential. Water influx is mainly mediated by putative lysosomal water channels (blue), and catabolite efflux is mediated by various carriers and transporters (brown). Prior to lysosome fission/reformation, solute and water efflux are required for osmotic condensation of endolysosomes or autolysosomes.

Lysosomes are acidic organelles containing more than 60 types of degradative hydrolases (Kolter and Sandhoff, 2005). Within the lysosome lumen, macromolecules such as proteins, polysaccharides, and complex lipids are digested into many more molecules of free amino acids, monosaccharides, and free fatty acids, resulting in a dramatic increase in the luminal osmolarity (Berg et al., 1994; Kolter and Sandhoff, 2005; see Fig. 1). These hydrolytic reactions require consumption of a large number of water molecules (Kolter and Sandhoff, 2005; see Fig. 1). For instance, complete proteolytic degradation of one molecule of protein with 100 amino acid (AA) residues requires 99 molecules of water (Fig. 1). As a result, although water concentration is only slightly reduced, water potential (see text box) is significantly decreased relative to the cytosol (Nobel, 2009). Hence, lysosomes must contain both osmo-sensing machinery and a water transport system to keep a relatively constant isotonic environment in the lumen. If there exists constitutive water permeability on the lysosomal membranes, water potential in the lumen can be quickly restored. Although the lipid bilayer is intrinsically water permeable at a certain degree, most cellular membranes may contain protein-based water permeability, e.g., those mediated by water channels (Agre et al., 2002; Fushimi et al., 1993). A limited water permeability on the lysosomal membranes would result in their lumens becoming hyperosmolar or hypertonic, at least transiently. If the catabolic degradation products are not quickly removed through catabolite exporters, the elevated osmolality may force excessive water influx into the lumen down the water potential gradient, resulting in increases in lysosomal volume (swelling) and membrane tension (Nobel, 2009; Saric and Freeman, 2020). Therefore, to maintain the osmotic balance, as well as lysosome morphology and homeostasis, both ion and catabolite channels/transporters must work cooperatively according to the osmo-status of luminal contents.

When catabolite transporters are unavailable, for instance, in the *Spinster* mutant in which sugar transport is defective (Rong et al., 2011), lysosomes are osmotically swollen (Fig. 1). Likewise, in cystinosis, a lysosomal storage disease (LSD) caused by mutations in the cystine transporter *CTNS*, cyst(e)ine accumulation in the lysosome leads to osmotic swelling (Festa et al., 2018; Kalatzis et al., 2001). Due to the lack of a sucrose transporter in lysosomes, sucrose that is experimentally delivered through the endocytic pathway accumulates in lysosomes to cause osmotic swelling (Cohn and Ehrenreich, 1969). In all cases, such swollen lysosomes are defective in degradation and trafficking (Bandyopadhyay et al., 2014). In addition, glycyl-*L*-phenylalanine 2-naphthylamide (GPN), an artificial substrate of lysosomal proteases, may induce osmotic swelling of lysosomes (Berg et al., 1994; Li et al., 2019). In this case, as the export of degradation products is not impaired, lysosomal swelling is limited and quickly reversible. Thus, efficient export of catabolites is necessary for osmotic homeostasis of lysosomes. When lysosomal hydrolases are mutated, as in many LSDs, abnormal accumulation of undigested materials causes lysosomal enlargement (Xu and Ren, 2015). There are two distinct causes underlying apparent lysosomal enlargement: (1) osmotic swelling and (2) trafficking defects due to excessive fusion or defective fission. In the cases of LSDs caused by enzyme defects, it is not clear whether lysosomal enlargement is due to osmotic swelling, trafficking defects associated with lysosomal storage, or both (Liu et al., 2012; Xu and Ren, 2015). As similar machineries may operate on lysosomal vs. plasma membranes, we first review the water and solute flux mechanisms that regulate cell size/volume.

### Plasmalemmal solute and water flux in cell volume regulation

**Figure 2.**
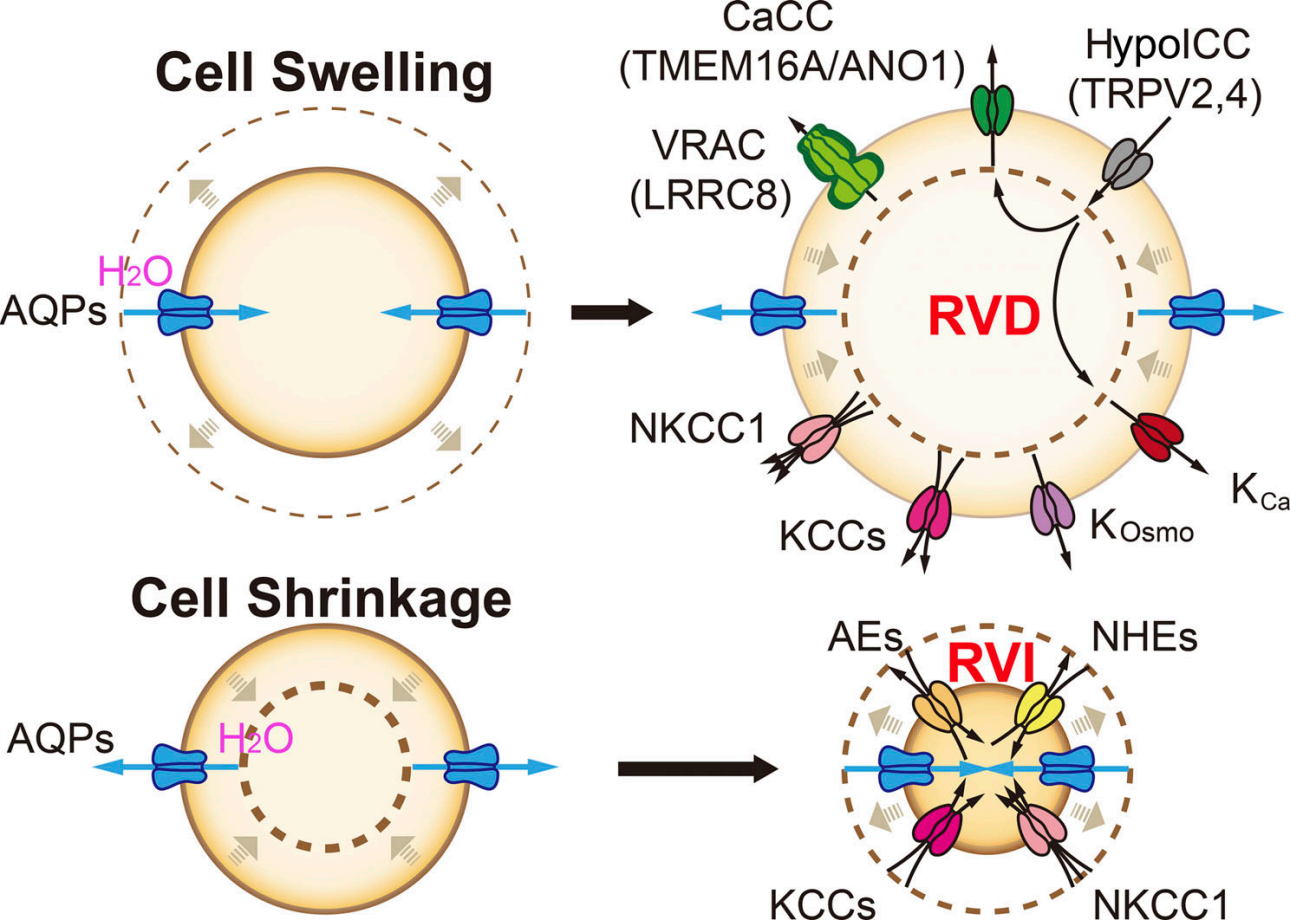
**Plasmalemmal ion and water transport in cell volume regulation.** Upon hypotonic and hypertonic challenges, cells undergo swelling and shrinkage, respectively. In order to maintain and restore the volume constancy for cellular metabolism, cells have evolved regulatory volume decrease (RVD) and increase (RVI) responses. During RVD, various K^+^ and Cl^−^ channels and transporters are activated in response to extracellular hypotonicity, which include VRAC (Cl^−^ and organic anions), CaCC (Cl^−^), osmo-sensitive and calcium-activated K^+^ channels (K^+^), H(ypo)ICCs (Ca^2+^), KCCs (K^+^ and Cl^−^), and NKCC1 (Na^+^, K^+^, and Cl^−^). Subsequently, osmotically obligated water efflux is mediated by AQPs. During RVI, various channels and transporters are activated in response to extracellular hypertonicity to transport solutes into cytosol, which include anion exchangers (Cl^−^ and HCO_3_^−^), NHEs (Na^+^ and H^+^), KCCs, and NKCC1. Osmotically obligated water influx is also mediated by AQPs.

When mammalian cells are bathed in hypertonic (high osmolality) or hypotonic (low osmolality) solutions, the water potential and osmotic pressure produced by the water and osmolyte concentration gradients drive water flux across membranes to cause cell shrinkage or swelling, respectively (Fig. 2). As the plasma membrane is incapable of stretching by more than 5%, an increase in cell volume by more than 10% could cause plasma membrane rupture and necrotic cell death (Ritter et al., 2021; Shubin et al., 2016). To maintain cellular homeostasis, RVD and RVI are triggered to readjust cell volume (Hoffmann et al., 2009; Fig. 2). During RVD, ions and organic osmolytes—such as amino acids, small carbohydrates, and their derivatives—are extruded from the cytoplasm, followed by water efflux (Ritter et al., 2021). Solute flux is triggered by the osmotic stimulus, either a change in the cytosolic osmolality or ionic strength (Strange et al., 2019; Syeda et al., 2016), while water flux is driven by a transmembrane difference in the water potential and is primarily mediated by aquaporin (AQP) water channels on the plasma membrane (Agre et al., 2002; King et al., 2004). The most abundant osmolytes in the cell are K^+^, Na^+^, and Cl^−^. Hence, during RVD, cell swelling may directly or indirectly activate K^+^ and Cl^−^ channels as the osmo-effectors; during RVI, cell shrinkage may directly or indirectly activate Na^+^ channels and K^+^/Cl^−^ transporters as the osmo-effectors (Hoffmann et al., 2009; Jentsch, 2016; Qiu et al., 2014; Russell, 2000; Voss et al., 2014; Wehner, 2006). While most ion channels or transporters involved in RVD and RVI are membrane-bound osmo-sensors themselves, some are stimulated indirectly by cytosolic osmo-sensors (Hoffmann et al., 2009; Jentsch, 2016; Okada et al., 2001; Shekarabi et al., 2017). In order to achieve electroneutrality, both Cl^−^ and K^+^ channels are activated simultaneously to provide the parallel conductance for each other during regulatory volume responses (Hoffmann et al., 2009; Jentsch, 2016; Wehner, 2006). Alternatively, additional symporters and exchangers/antiporters are recruited to provide the parallel conductance (Hoffmann et al., 2009; Jentsch, 2016).

#### Osmo-regulated Cl^−^ channels

Volume-regulated anion channels (VRACs) play a key role in RVD by mediating the eviction of Cl^−^ and organic osmolytes during hypotonic stress (Qiu et al., 2014; Voss et al., 2014; Fig. 2). VRACs are composed of LRRC8 family proteins; the essential subunit LRRC8A/SWELL1 forms a hexameric complex with other subunits, including LRRC8B-E (Qiu et al., 2014; Voss et al., 2014). This subunit composition defines the pore properties in anion permeability/selectivity, channel activation and inactivation, rectification, and single-channel conductance, which in turn determine the kinetics of Cl^−^ or anion flux (König and Stauber, 2019; Syeda et al., 2016). VRAC activation is evoked during cell swelling, e.g., reduced cytosolic osmolality or ionic strength, within a few seconds to minutes (Jentsch, 2016; Qiu et al., 2014; Syeda et al., 2016). Other hypotonicity-induced changes, such as generation of reactive oxygen species (ROS), may also regulate VRAC activation (Gradogna et al., 2017; Jentsch, 2016). Upon activation, VRAC is permeable to Cl^−^, as well as some organic solutes such as glutamate, taurine, myo-inositol, and ATP (Jackson and Strange, 1993; Kirk et al., 1992). In some cell types, Cl^−^ channels other than VRAC may also be activated, directly or indirectly by hypotonicity. For instance, TMEM16A/ANO1, a Ca^2+^-activated Cl^−^ channel (CaCC), is activated by membrane stretch associated with cell swelling and/or through cytosolic Ca^2+^ increases (Benedetto et al., 2016) through hypotonicity-induced cation channels (HypoICCs), e.g., TRPV2 and TRPV4 (Liedtke, 2000; Sato et al., 2013; Fig. 2).

#### Osmo-regulated K^+^ channels

As the parallel conductance to Cl^−^ through VRACs, osmo-regulated K^+^ channels mediate K^+^ efflux during RVD (Hoffmann et al., 2009; Fig. 2). Swelling-induced K^+^ channels include TREK-1/KCNK2, KCNQ/Kv7/M-channel, and TASK-2/KCNK5 (Jentsch, 2016; Kirkegaard et al., 2010). Notably, K^+^ channels may be activated indirectly by cell swelling. Upon swelling-induced cytosolic Ca^2+^ increase through HypoICCs, various Ca^2+^-dependent, voltage-sensitive K_Ca_ channels, i.e., BK, IK, SK channels, may mediate K^+^ efflux (Latorre et al., 2017). Regardless of the participating channels and their activation mechanisms, the decrease of cytosolic [K^+^] caused by K^+^ efflux contributes to RVD by creating an osmotic force and water potential gradient to extrude the water from the cytosol.

#### Osmo-regulated ion transporters

During RVD and RVI, various K^+^ and Cl^−^ transporters, including Na^+^-K^+^-2Cl^−^ cotransporter (NKCC1) and K^+^-Cl^−^ cotransporters (KCCs), may couple K^+^ transport with Cl^−^ transport, or vice versa, to achieve an electro-neutral net solute flux (Boettger et al., 2002; Jentsch, 2016). Ion transporters are preferentially activated during RVI (Hoffmann et al., 2009; Jentsch, 2016). For example, NKCC1 and KCCs, as well as Na^+^-H^+^ exchangers (NHEs), epithelial Na^+^ channels (ENaCs), and Cl^−^-HCO_3_^−^ anion exchangers (AEs), are all stimulated by hypertonic stress, resulting in influx of Na^+^, K^+^, and Cl^−^ (Plettenberg et al., 2008; Ritter et al., 2021; Wehner, 2006; see Fig. 2). Stimulated by extracellular hypertonic stress or cell shrinkage, cytoplasmic osmo-sensors, e.g., WNK-SPAK/OSR1 kinase, may phosphorylate KCCs to increase K^+^ and Cl^−^ influx (de Los Heros et al., 2006; Garzón-Muvdi et al., 2007; Jentsch, 2016; Shekarabi et al., 2017). The SLC26 gene family encodes multifunctional anion exchangers that transport a wide range of osmolytes, including Cl^−^ and HCO_3_^−^ (Alper and Sharma, 2013; Fig. 2). Upon hypertonic stimulation, SLC26 transporters may traffic from intracellular compartments to the plasma membrane (Xu et al., 2006). Therefore, ion transporters may be upregulated in activity or expression to promote solute flux during regulatory cell volume responses.

#### Organic osmolyte flux mechanisms

Small organic solutes, such as polyols (sorbitol and myo-inositol), amino acids, and derivatives (glutamate, aspartate, glycine, proline, alanine, and taurine; Garcia-Perez and Burg, 1991), are exported from the cells during RVD through specific carbohydrate and amino acid transporters (Liu et al., 2012; Sagne et al., 2001; Wyant et al., 2017). In addition, LRRC8C/LRRC8E-containing VRACs are permeable to negatively charged amino acids such as aspartate and glutamate, and LRRC8D-containing VRACs are permeable to a range of organic osmolytes (Schober et al., 2017). Hence, during RVD, osmo-sensitive K^+^ and Cl^−^ channels/transporters, as well as organic solute channels/transporters, are all simultaneously activated in order to quickly release osmolytes (Ritter et al., 2021).

#### Water channels and AQPs

The efflux of osmolytes produces an osmotic pressure, and simultaneously an outward water potential gradient, to facilitate passive water efflux. Although there is a slow, limited water permeability through passive diffusion across lipid bilayers, most transmembrane water flux is through facilitated diffusion mediated by the AQPs (Agre et al., 2002; Olesen and Fenton, 2021; Preston et al., 1992; Zeuthen, 2010). There are 13 isoforms of AQPs in humans (Day et al., 2014), most of which are constitutively active and selectively water permeable (Fig. 2). However, cytosolic signaling may regulate the surface expression of AQPs through vesicular trafficking (Noda and Sasaki, 2005). In the kidney tubules, the asymmetric distribution of solute channels/transporters vs. AQPs in the Loop of Henle is responsible for dilution or condensation of tubular lumen in distinct segments (Nielsen et al., 2002). Whether there exist dedicated primary water transporters that drive water flux uphill the water potential gradient is not known. However, water can be co-transported, either uphill or downhill, in some secondary transporters (Zeuthen, 2010), and VRACs exhibit certain water permeability (Nilius, 2004). Since VRACs activation, and possibly AQPs redistribution, is triggered by cell swelling, water transport across membranes may be upregulated under conditions such as hypotonic stress.

### Ion flux mechanisms in the lysosome

**Figure 3.**
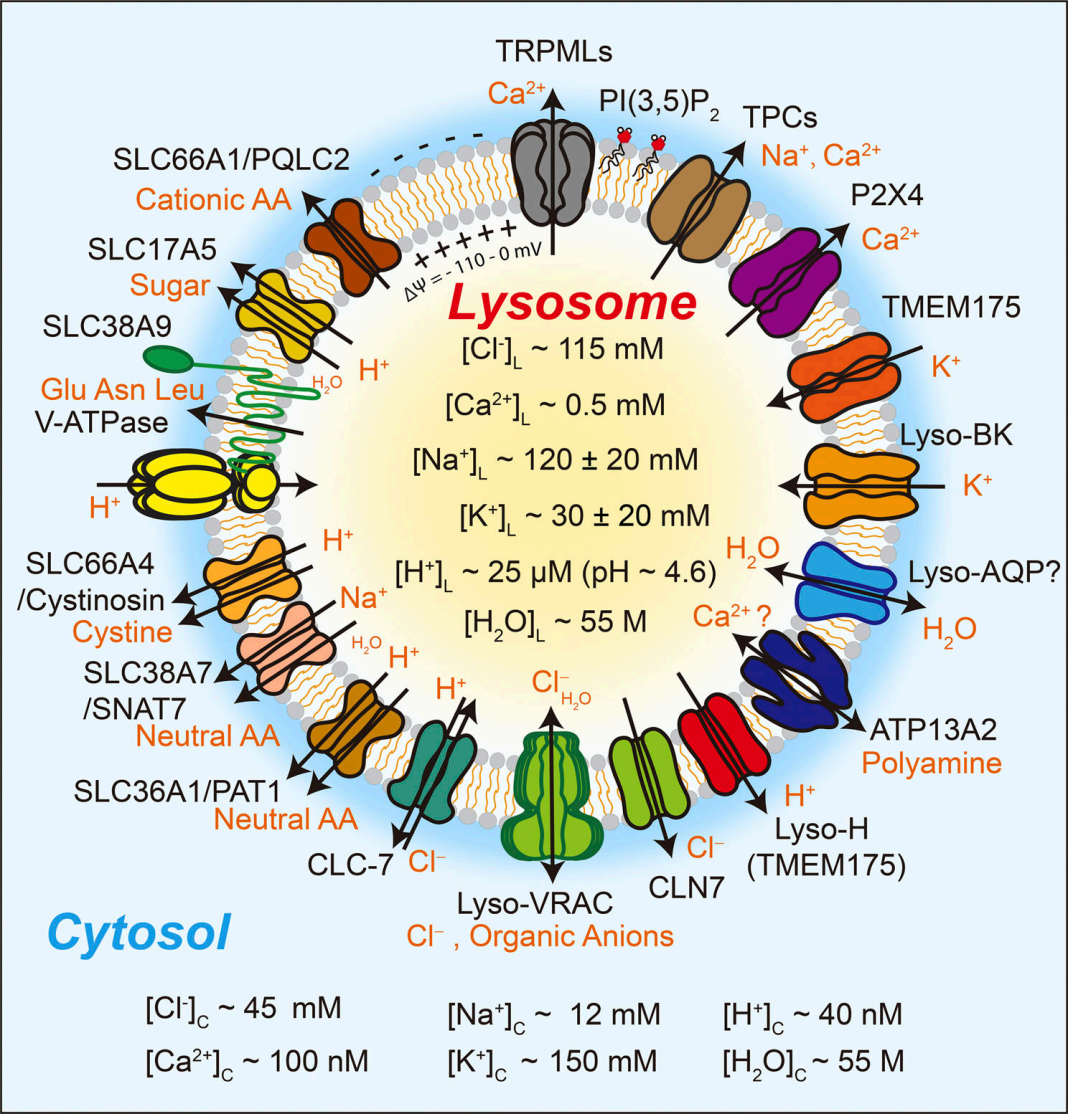
**Lysosomal ion channels and catabolite exporters Relative to the cytosol, lysosome lumen is high in [H**^**+**^**], [Ca**^**2+**^**], and [Na**^**+**^**], but low in [K**^**+**^**].** Lysosomal patch-clamp studies have identified lysosomal Ca^2+^-permeable TRPMLs and P2X4 channels, H^+^/K^+^-permeable TMEM175 channels, Na^+^/Ca^2+^-permeable TPC channels, K^+^-permeable Lyso-BK and TWIK2 channels, and Cl^−^-permeable CLC7 transporters, CLN7 channels, and Lyso-VRAC channels. The proton pump V-ATPase and LysoH/TMEM175 channels regulate lysosomal acidity. Catabolite efflux is mediated by SLC family transporters, which include carbohydrate transporter SLC17A5 (H^+^-coupled), cationic amino acid (AA) uniporter SLC66A1/PQLC2, polar AA (Glu/Asn/Leu) transporter SLC38A9 (H^+^-coupled), cystine transporter SLC66A4/Cystonisin (H^+^-coupled), and neutral AA transporters SLC38A7/SNAT7 (Na^+^-coupled) and SLC36A1/PAT1 (H^+^-coupled). ATP13A2 may mediate polyamine export and possibly Ca^2+^ import. Water flux across the lysosomal membrane is mediated by unidentified water channels (Lyso-AQPs), Lyso-VRAC, and possibly certain catabolite transporters.

Lysosomal ion channels and transporters regulate and use the concentration gradients of H^+^, Na^+^, K^+^, Ca^2+^, and Cl^−^ across lysosomal membranes (Fig. 3; Li et al., 2019; Xu and Ren, 2015). Lysosomal Δψ (= ψ_cytosol_−ψ_lumen_), determined by the relative permeability of monovalent ions such as Na^+^, K^+^, H^+^ and Cl^−^, and divalent ions such as Ca^2+^ (Cao et al., 2015b; Li et al., 2019; Saminathan et al., 2021; Wang et al., 2017), was estimated to be −110 to 0 mV in the cells (Koivusalo et al., 2011; Saminathan et al., 2021). As the lysosome lumen is high in [Na^+^], [H^+^], [Ca^2+^], and [Cl^−^], but low in [K^+^], relative to the cytosol (Leung et al., 2019; Narayanaswamy et al., 2019; Steinberg et al., 2010; Wang et al., 2012), the electrochemical gradients favor lumen-to-cytosol efflux of Na^+^, H^+^, and Ca^2+^. Lysosomal ionic composition and ion flux have important roles in regulating lysosomal functions including membrane fusion and fission, as well as osmotic swelling and condensation of lysosomes (Li et al., 2019; Luzio et al., 2007b). For instance, lysosomal Ca^2+^ release, acting through Ca^2+^ effectors such as Calmodulin (CaM) and ALG-2, regulates solute flux as well as membrane fusion and fission (Cao et al., 2015a; Li et al., 2016). Likewise, activation of specific ion-selective channels in the lysosomes may release Na^+^ from the lumen to the cytosol, but uptake K^+^ from the cytosol into the lumen, contributing to osmotic regulation of lysosome volume (Wang et al., 2017; Wang et al., 2012; Xu and Ren, 2015). Lysosomal Δψ may regulate both catabolite export and membrane trafficking (Sagne et al., 2001; Xiong and Zhu, 2016). Lysosomal ion channels are regulated by various nutrient-dependent and trafficking-related cellular signals (Li et al., 2019). Hence, while trafficking cues may regulate membrane fusion and fission through lysosomal ion channels, osmotic cues may regulate organelle volume through lysosomal channels as well.

#### H^+^ and TMEM175/LyPAP

Most lysosomal hydrolases require an acidic lumen for their optimal activities (Mellman, 1989). Lysosomes establish and maintain more than 500-fold [H^+^] gradient across their membranes through a proton-pumping V-ATPase (Mindell, 2012; Fig. 3). The H^+^ gradient may drive solute flux through H^+^-coupled ion and catabolite transporters (Kalatzis et al., 2001; Sagne et al., 2001). Lysosomal H^+^ release is proposed to regulate sorting such as ESCRT-dependent inward budding and formation of intraluminal vesicles, as well as mobility and membrane trafficking of lysosomes (Li et al., 2019; Luzio et al., 2007b). Luminal H^+^ also regulates the activities of various lysosomal ion channels (Cang et al., 2014; Hu et al., 2022; Li et al., 2019; Xu et al., 2007). Because inhibition of V-ATPase leads to rapid lysosomal de-acidification, there must exist an unidentified proton leak conductance (LysoH) on lysosomal membranes (Christensen et al., 2002; Xiong and Zhu, 2016; Xu and Ren, 2015). Because the LysoH current is absent in TMEM175 KO cells, but dramatically increased upon TMEM175 overexpression, TMEM175 is likely the molecular basis of LysoH (Hu et al., 2022; Zheng et al., 2022). Acting as a proton-activated proton channel (LyPAP), TMEM175 may regulate the lysosomal pH set-point and optimum to prevent lysosomal over-acidification (Hu et al., 2022; Zheng et al., 2022). However, a complete inhibition of V-ATPase in TMEM175 KO cells still leads to lysosomal de-acidification, suggesting the existence of additional H^+^ leak pathways, especially within the less acidic pH ranges (Hu et al., 2022). H^+^-coupled ion transporters, e.g., CLC7 (Graves et al., 2008), and H^+^-coupled catabolite transporters (Kalatzis et al., 2001; Sagne et al., 2001), may mediate such slow H^+^ leak or efflux pathways. It remains to be determined whether TMEM175 and transporter-mediated slow H^+^ leak regulate the above-mentioned H^+^-dependent lysosomal trafficking.

#### Ca^2+^, TRPMLs, and P2X4

Lysosomes are mobile intracellular Ca^2+^ stores with luminal [Ca^2+^] ∼0.5 mM, 5,000-fold higher than cytosolic [Ca^2+^] (Yang et al., 2019; Fig. 3). ATP13A2, a candidate Ca^2+^ transporter, or still unidentified Ca^2+^ transporters might mediate Ca^2+^ import (Narayanaswamy et al., 2019; van Veen et al., 2020). Lysosomal Ca^2+^ efflux may regulate a variety of lysosomal functions, including membrane fusion and fission (Cao et al., 2017; Cao et al., 2015a; Li et al., 2016; Xu and Ren, 2015). BAPTA, a fast Ca^2+^ chelator, but not EGTA, a slow Ca^2+^ chelator, blocks lysosome fusion with other organelles and plasma membrane in vitro and in vivo, suggesting that lysosomal Ca^2+^ release is a key regulator of lysosomal fusion (Luzio et al., 2007a; Peters and Mayer, 1998; Pryor et al., 2000; Samie et al., 2013). In addition, lysosomal Ca^2+^ release may also regulate membrane fission and content condensation (Cao et al., 2017; Luzio et al., 2007a; Saffi and Botelho, 2019; Zou et al., 2015). Transient receptor potential cation channels of the mucolipin subfamily 1–3 (TRPML1-3) are the primary lysosomal Ca^2+^ release channels (Zhang et al., 2018; Fig. 3). TRPMLs are activated by PI(3,5)P_2_, a lysosome-localized phosphoinositide generated by the kinase PIKfyve (Dong et al., 2010; Zolov et al., 2012). PI(3,5)P_2_ regulates various aspects of lysosome function, including membrane fusion/fission and solute transport (Dong et al., 2010; Rivero-Ríos and Weisman, 2022). TRPML1 is also activated by ROS to cause nuclear translocation of transcriptional factor EB (TFEB), a master regulator of lysosome biogenesis (Zhang et al., 2016). TRPML1 deficiency causes endolysosomal vacuolation in the cells, suggesting that TRPML1 regulates membrane trafficking and/or solute transport (Dayam et al., 2015; Pryor et al., 2000). Additional lysosomal Ca^2+^ release may be mediated by P2X4 channels, or by TPCs in an agonist-specific manner (Calcraft et al., 2009; Cao et al., 2015a; Gerndt et al., 2020; Li et al., 2019; Fig. 3). Activation of TPCs’ Ca^2+^ conductance by NAADP may regulate lysosomal membrane fusion and trafficking (Vassileva et al., 2020). Overexpression of P2X4 channels reportedly increases lysosome size (Cao et al., 2015a), suggesting that P2X4 regulates Ca^2+^-dependent lysosomal fusion.

#### Na^+^ and TPCs

Luminal [Na^+^] is higher than cytosolic [Na^+^] (Steinberg et al., 2010; Wang et al., 2012; Fig. 3), although the ion transporters that establish the lysosomal Na^+^ gradient are not yet known. When extracellular Na^+^ is replaced with non-permeant cations (e.g., NMDG^+^), shrinkage of macropinosomes is prevented, suggesting that Na^+^ release is required for content condensation of endosomes, and possibly lysosomes (Freeman et al., 2020). TPC channels, TPC1 and TPC2, are highly Na^+^-selective channels (P_Ca_/P_Na_ ∼0.1) in the lysosomes, although their Ca^2+^ permeability may become significant (P_Ca_/P_Na_ ∼0.7) if the channels are activated by NAADP and certain synthetic agonists (Brailoiu et al., 2009; Calcraft et al., 2009; Gerndt et al., 2020; Wang et al., 2012; Yuan et al., 2022; Zong et al., 2009; Fig. 3). On the other hand, PIKyve-generated PI(3,5)P_2_ can specifically activate TPCs’ Na^+^ conductance (Dong et al., 2010; She et al., 2018; Wang et al., 2012). While PIKfyve kinase activity is stimulated by osmotic stress, pharmacological inhibition of PI(3,5)P_2_ production causes endolysosomal vacuolation in mammalian cells (Cai et al., 2013; Sharma et al., 2019). It is possible that PI(3,5)P_2_ regulates lysosomal membrane trafficking and Na^+^-release-dependent condensation through TPCs. However, in TPC1 and TPC2 double knockout cells (TPC DKO), inhibition of PIKfyve can still cause lysosomal enlargement (Zhang et al., 2019b), suggesting that TPCs may mediate the effects of PI(3,5)P_2_ on solute flux, but not on membrane fusion/fission. While TPC1 may regulate PI(3,5)P_2_-dependent Na^+^ release from macropinosomes, mediating condensation of macropinosomes, it is hypothesized that TPC2 channels may play a similar role in lysosomal content condensation (Freeman et al., 2020).

#### K^+^, LysoK_VCa_, and TMEM175

[K^+^] is high in the cytosol, but low in the lumen (Fig. 3; Li et al., 2019; Steinberg et al., 2010; Wang et al., 2012). LysoK_V__Ca_/Lyso-BK, TMEM175, and TWIK2 are K^+^ channels in the lysosomes that can mediate K^+^ flux cross-lysosomal membrane (Bobak et al., 2017; Cang et al., 2015; Wang et al., 2017). Note that TMEM175’s K^+^ conductance is suppressed at lysosomal pH (Hu et al., 2022; Zheng et al., 2022). In addition, activation of lysosomal K^+^ channels may cause changes in lysosomal Δψ, which may in turn regulate TRPML1-mediated Ca^2+^ release, the refilling of lysosomal Ca^2+^ stores, hence Ca^2+^-dependent lysosomal functions (Wang et al., 2017; Wang et al., 2021), and potentially V-ATPase-mediated H^+^ import (Mindell, 2012). It remains to be investigated whether lysosomal K^+^ channels play a role in the osmotic swelling of lysosomes.

#### Cl^−^ and Lyso-VRAC

Luminal [Cl^−^] is estimated to be ∼115 mM, which is several fold higher than cytosolic [Cl^−^] (∼45 mM, Fig. 3; Chakraborty et al., 2017) and the Cl^−^ equilibrium potential could be approximately −20 mV (cytosolic negative) under resting state. Hence, the direction of lysosomal Cl^−^ flux may be dependent on lysosomal Δψ. Cl^−^ flux may in turn regulate lysosomal Δψ, and subsequently Ca^2+^ release (Chakraborty et al., 2017; Wang et al., 2021). Three types of Cl^−^ channels/transporters are known to be present in the lysosomes. CLC7, a Cl^−^/H^+^ exchanger, might provide counter ions to support lysosomal acidification (Graves et al., 2008; Mindell, 2012; but also see Weinert et al., 2010; Fig. 3). CLN7/MFSD8, which is mutated in neuronal ceroid lipofusinose (NCL), is recently reported to mediate a lysosomal Cl^−^ conductance (Wang et al., 2021). LRRC8 family proteins form lysosomal volume-regulated anion channels (Lyso-VRACs) that are activated by cytosolic hypotonicity or low ionic strength (Li et al., 2020; Fig. 3). Through a lysosome-targeting motif in LRRC8A, LRRC8 proteins localize to lysosomes to constitute Lyso-VRAC (Li et al., 2020). Like plasma membrane VRAC, Lyso-VRAC is permeable to Cl^−^, as well as other anions such as glutamate and HCO_3_^−^ (Jackson and Strange, 1993; Li et al., 2020). Depending on Δψ and chemical gradients, Lyso-VRAC activation may lead to flux of these anionic solutes, in addition to Cl^−^ flux.

### Regulation of lysosome size: Mmembrane fusion and fission

Lysosome membrane fusion may serve two purposes: delivery of cargo or hydrolases and expansion of lysosome volume and size. Likewise, outward membrane fission can serve two purposes: retrieval or export of degradation products and lysosome resolution or reformation (Luzio et al., 2007b; Saffi and Botelho, 2019). Additionally, inward budding to form intraluminal vesicles may also regulate membrane remodeling and sorting (Babst, 2011; Hurley, 2008; Piper and Katzmann, 2007).

**Figure 4.**
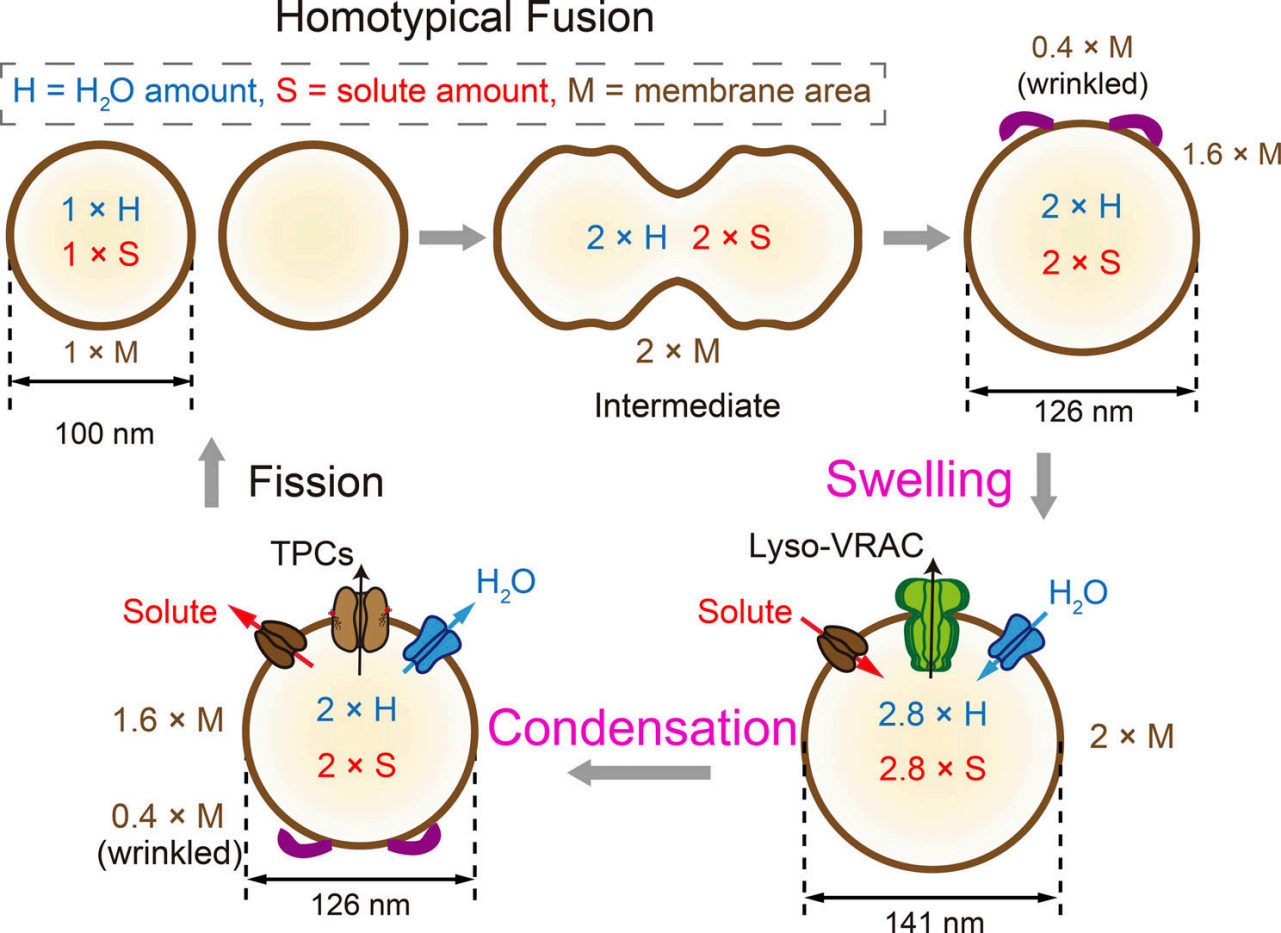
**Lysosome volume/size regulation: swelling and condensation, Homotypic fusion of two quasi-spherical lysosomes results in a larger quasi-spherical lysosome.** In the absence of water and solute efflux, there is ∼20% excess folded membrane in the newly formed lysosome, i.e., wrinkled membrane with low membrane tension. Upon solute flux, which could be stimulated by fusion cues and/or changes in membrane curvature through yet-to-be-identified solute channels/transporters, followed by subsequent water flux, the newly formed lysosome is enlarged to contain additional ∼40% volume, i.e., turgid membrane with high membrane tension. Upon TPC-mediated Na^+^ release and subsequent water flux, the enlarged lysosome shrinks to reduce the volume and surface area (membrane).

**Figure 5.**
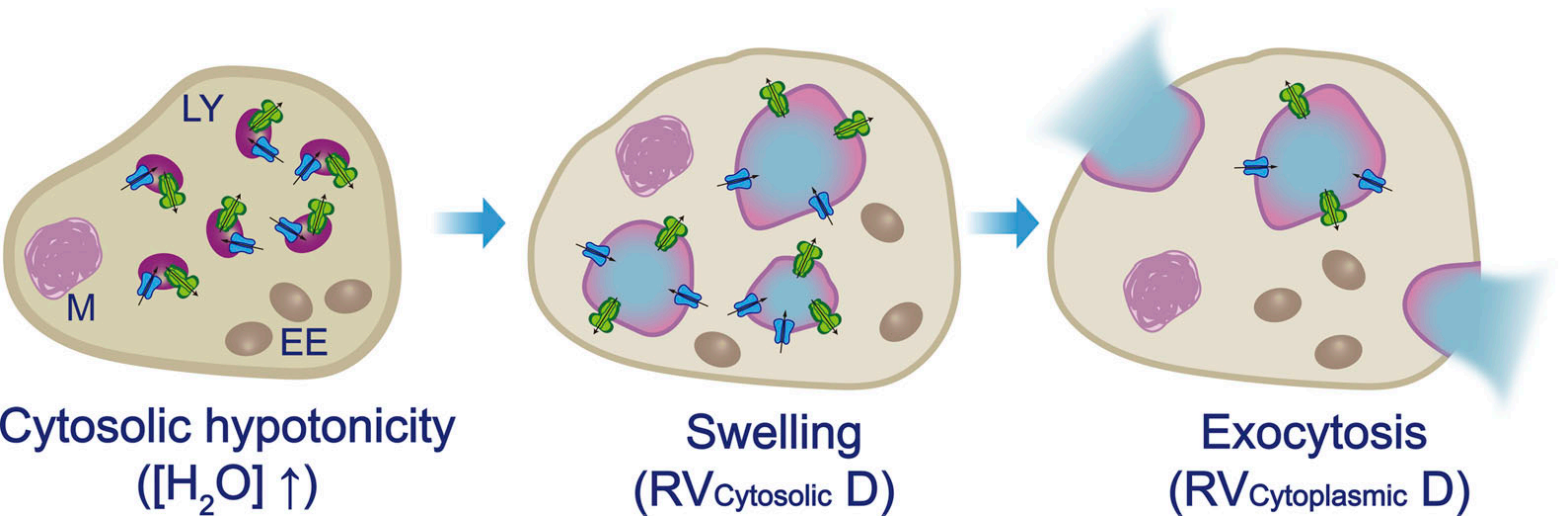
**Lysosomes are water-storage organelles that mediate exocytosis-dependent water secretion.** When mammalian cells are exposed to a hypotonic environment, cytosolic [H_2_O] increases rapidly through plasma membrane AQPs. Lysosomes, but not early endosomes or mitochondria, selectively uptake water. Cytoplasmic volume = cytosolic volume + organellar volume. By acting as intracellular water storage compartments, lysosomes contribute to cytosolic volume decrease (cytosolic RVD). Upon exocytosis of these watery lysosomes, cytoplasmic volume is decreased (cytoplasmic RVD).

Primary lysosomes receive cargo macromolecules from endosomes, phagosomes, and autophagosomes through membrane fusion. There are three consecutive steps during membrane fusion: tethering, SNARE complex formation, and lipid bilayer mixing (Advani et al., 1999; Luzio et al., 2007b; Prekeris et al., 1999; see Figs. 4 and 5). Lysosomal Ca^2+^ release may regulate these fusion steps, similar to Ca^2+^ regulation of synaptic vesicle exocytosis (Cao et al., 2017; Li et al., 2019; Fig. 4). Lysosomal Ca^2+^ channels, including TRPML1, P2X4, and TPCs, are proposed to regulate Ca^2+^-dependent lysosome fusion with late endosomes and autophagosomes (Cao et al., 2015a; Li et al., 2019; Ruas et al., 2010; Scotto Rosato et al., 2019; Fig. 4). TRPML1-mediated Ca^2+^ release is increased when lipidated LC3 proteins bind to TRPML1 upon autophagosome-lysosome fusion (Nakamura et al., 2020). In some specialized cell types, TRPML2 and TRPML3 may also regulate Ca^2+^-dependent lysosomal membrane fusion (Dong et al., 2010; Li et al., 2019; Zhang et al., 2019a). As TRPML2 is also sensitive to hypotonicity, lysosomal Ca^2+^ release may play an important role in osmo-regulation of membrane trafficking (Chen et al., 2020). Dual regulation of TRPMLs by trafficking cues, such as PI(3,5)P_2_, and osmotic cues, suggests that membrane trafficking and solute transport are interconnected. Lysosomal Na^+^ release and changes in lysosomal Δψ are also proposed to promote membrane fusion, but direct evidence is still lacking (Wang et al., 2012).

Lysosome membrane fission is required for retrograde lysosome-to-TGN membrane trafficking and reformation of lysosomes from autolysosomes, phagolysosomes, and endolysosomes (Ripoll et al., 2018; Saffi and Botelho, 2019). The fission process requires coat and adaptor proteins, and is regulated by motor protein-driven vesicle movement (Ripoll et al., 2018; Saffi and Botelho, 2019). Fission steps are regulated by trafficking cues; for example, lipid signals such as PI(3,5)P_2_ and PI(4,5)P_2_ may help recruit the fission machinery or regulators (Chen and Yu, 2017; Rong et al., 2012). While pharmacological inhibition of PIKfyve impairs endolysosome fission and retrograde trafficking, washout of PIKfyve inhibitors results in lysosomal tubulation and vesiculation (Freeman et al., 2020; Saric and Freeman, 2020; Sharma et al., 2019). PI(3,5)P_2_ may control membrane remodeling by recruiting fission players to the lysosomal membranes, or by activating lysosomal channels (Saric and Freeman, 2020). Importantly, both PI(3,5)P_2_-regulated TRPML1-mediated Ca^2+^ release and TPC-mediated Na^+^ release are reported to regulate lysosome mobility, tubulation, and fission (Cao et al., 2017; Li et al., 2019; Li et al., 2016; Saric and Freeman, 2020).

### Regulation of lysosome size: Swelling and condensation

Upon hypotonic shock, lysosomes become swollen (Groulx et al., 2006; Iwasa et al., 2001; Li et al., 2020). The membranes of such hypotonicity-induced swollen lysosomes are more fragile than the lysosomes enlarged due to excessive fusion or impaired fission (Li et al., 2020). Such osmotic swelling can be seen in isolated native lysosomes or chemically enlarged lysosomes (Li et al., 2020). Hence, osmotic swelling of lysosomes (without membrane addition) is different from lysosome enlargement caused by unbalanced membrane trafficking (with membrane addition). While lysosomal swelling and fusion may both increase the size of lysosomes, membrane tension is increased by osmotic swelling resulting in a turgid membrane but reduced following fusion causing a wrinkled membrane. As increased membrane tension may trigger further membrane fusion as a negative feedback compensatory mechanism (Li et al., 2020; Saric and Freeman, 2020), osmotic swelling may indirectly promote membrane fusion.

Reformation of proto-lysosomes from secondary lysosomes upon completion of lysosomal degradation requires condensation of the luminal contents as a prerequisite step for fission, in order to reduce membrane tension so that membrane deformation is permitted (Luzio et al., 2007b). Solute/water efflux is required for such condensation, which is known to be dependent on luminal H^+^ and Ca^2+^ (Pryor et al., 2000). While luminal acidification may be required for catabolite export, Ca^2+^ may regulate solute transport or fission machinery (Cao et al., 2017; Li et al., 2019; Li et al., 2016; Saric and Freeman, 2020). Thus, osmotic regulation of solute transport and lysosomal channels may have an indirect effect on membrane fission.

#### Solute and water transport in the lysosome

Lysosomal Na^+^, K^+^, and Cl^−^ channels may all contribute to solute transport across lysosomal membranes (Fig. 4). Among them, TPCs may mediate the release of Na^+^ from the lumen (Saminathan et al., 2021; Steinberg et al., 2010; Wang et al., 2012), contributing to lysosomal condensation (Freeman et al., 2020). Likewise, lysosomal Cl^−^ channels/transporters such as Lyso-VRAC may release luminal Cl^−^, contributing to condensation (Li et al., 2020). PAC/ASOR/TMEM206, a H^+^-activated Cl^−^ channel, is shown to mediate H^+^-dependent micropinosomal Cl^−^ efflux and shrinkage (Zeziulia et al., 2022). Paradoxically, Lyso-VRAC is required for hypotonicity-induced lysosomal swelling, suggesting that Lyso-VRAC may also mediate solute and/or water influx under hypotonic stress (Li et al., 2020). LysoK_VCa_ and other lysosomal K^+^ channels may help import K^+^, potentially involved in swelling (Cang et al., 2015; Wang et al., 2017). On the other hand, non-selective channels such as TRPMLs may not be effective for net solute transport because their reversal potential is close to lysosomal Δψ (Li et al., 2019). In addition, luminal H^+^ and Ca^2+^, given its low concentration, may not be an effective solute for transport. However, lysosomal H^+^ and Ca^2+^ channels may regulate the solute transporters indirectly through juxta-lysosomal H^+^ and Ca^2+^, or lysosomal Δψ regulation (Li et al., 2019; Wang et al., 2021).

Catabolite transporters may help release lysosomal solutes, contributing to condensation. Upon digestion of macromolecules into monomers at acidic luminal pH, these solutes are released from lysosomes through catabolite exporters. Notably, the lysosome lumen contains a large amount of osmolytes, including amino acids and their derivatives, whose transporters are not yet identified in many cases (Adelmann et al., 2020; Kalatzis et al., 2001; Liu et al., 2012; Sagne et al., 2001; Verdon et al., 2017; Wang et al., 2015). Impaired solute export may cause osmotic swelling (Bandyopadhyay et al., 2014; Li et al., 2020; Xu and Ren, 2015). The lysosomal organic solutes are mainly transported by the solute carrier (SLC) protein families (Fig. 3). For example, SLC17A5 is a H^+^-driven sugar transporter, whose loss-of-function mutations cause Salla disease, in which accumulated sialic acids in lysosomes cause lysosomal swelling (Renlund et al., 1986; Fig. 3). Likewise, lysosomal amino acid transporters, which include the cystine transporter SLC66A4/Cystinosin, the small neutral amino acids transporter SLC36A1/PAT1, the cationic amino acids transporter SLC66A1/PQLC2, the sodium-coupled neutral amino acid transporters SLC38A7, the glutamate/asparagine/leucine transporter SLC38A9 (Wyant et al., 2017), may also contribute to solute transport (Liu et al., 2012; Sagne et al., 2001; see Fig. 3). Loss of lysosomal amino acid transporters causes osmotic swelling of lysosomes (Liu et al., 2012; Saric and Freeman, 2020).

Both lysosomal swelling and condensation may require a lysosomal water transport system. Because isolated lysosomes become quickly swollen in a hypotonic medium, there may exist water-permeable conductance/channels on the lysosomes (Li et al., 2020). However, the molecular basis of lysosomal water permeability is not known. Although most AQPs are localized at the plasma membrane (Agre et al., 2002), AQP-11 and AQP-12 are known to be “subcellular aquaporins” and localized in intracellular membranes such as endoplasmic reticulum (ER; Ishibashi et al., 2021). More importantly, AQP-6 was found to be present on V-ATPase-positive compartments, and up-regulated by acidic pH (Nielsen et al., 2002; Yasui et al., 1999a; Yasui et al., 1999b). Hence, the putative Lyso-AQPs may regulate lysosomal water flux through its lysosomal expression. In addition, Lyso-VRACs and certain catabolite transporters may exhibit a certain degree of water permeability (Nilius, 2004; see Fig. 3). Indeed, lysosomes isolated from Lyso-VRAC-inhibited or -depleted cells are less likely to be swollen, suggesting that Lyso-VRAC is water-permeable (Li et al., 2020). Notably, water may be co-transported uphill with catabolites through these water-permeable channels and transporters (Zeuthen, 2010). Introducing synthetic water channels to the lysosomes dramatically increases the osmotic tension of lysosomes (Lucherelli et al., 2021), suggesting the basal water permeability of lysosomes is likely low. It is likely there exist some basal water permeability in the lysosome, and osmotic stimulation may upregulate its activity. However, investigating the regulatory mechanisms of lysosomal water permeability may require the identification of the water-permeable channels and transporters.

#### Regulation of ion flux as a means to induce lysosomal swelling and condensation

Osmotic swelling of lysosomes is caused by solute and water influx, and condensation is mediated by solute and water efflux. Therefore, the cellular conditions that cause sustained activation of lysosomal solute flux pathways may provide an osmotic regulation of lysosome size. Low osmolality or ionic strength in the cytosol activates Lyso-VRAC, increasing Cl^−^ or anion flux (Li et al., 2020). Depending on the flux direction, the mechanisms that regulate Lyso-VRAC activity may contribute to swelling or condensation (Fig. 4). On the other hand, activation of lysosomal K^+^ channels is likely to cause cell swelling due to K^+^ influx (Fig. 4). As LysoK_V__Ca_/Lyso-BK is activated by juxta-lysosomal Ca^2+^ increase (Cao et al., 2015b; Wang et al., 2017), activation of lysosomal Ca^2+^ release may also cause lysosome swelling through Ca^2+^-dependent ion flux.

Lysosomal condensation may be mediated by activation of solute efflux channels. Lysosomes are enlarged in PI(3,5)P_2_-deficient cells (Dong et al., 2010; Ikonomov et al., 2001; Sharma et al., 2019). PI(3,5)P_2_ is proposed to activate TPCs to induce lysosome condensation, similar to micropinosome resolution (Freeman et al., 2020; Fig. 4). Consistently, overexpression of TPCs reportedly causes lysosomal tubulation (Freeman et al., 2020). However, lysosomes are enlarged by PIKfyve inhibition even in TPC DKO cells (Zhang et al., 2019b). PI(3,5)P_2_ is required for membrane fission or lysosome reformation, which may be TPC-independent, but TRPML1-dependent (Sharma et al., 2019). Therefore, PI(3,5)P_2_ may regulate both membrane fusion/fission and osmotic condensation through lysosomal channels. Notably, a block in condensation may also impair membrane tubulation and fission (Freeman et al., 2020). To separate the roles of TPCs in PI(3,5)P_2_-dependent fission vs. condensation, it is necessary to study the effects of small-molecule activation of TPCs (Zhang et al., 2019b) and PI(3,5)P_2_-insensitive mutant TPCs (She et al., 2018).

### Lysosomes as potential water-storage organelles

When unicellular protists such as social amoeba are facing hypotonic challenges, excess water enters the cytosol and then contractile vacuoles (CVs), which serve as the intracellular water storage compartments that expel water to the extracellular space through periodical Ca^2+^-dependent exocytosis (Fountain et al., 2007). Similar mechanisms may also operate in mammalian cells, as CV-like large cytoplasmic vacuoles appear quickly when cells are facing hypotonic challenges (Jaiswal et al., 2019; King et al., 2020; Li et al., 2020). It is recently shown that membranes and luminal contents of these watery vacuoles are mostly derived from lysosomes (Li et al., 2020), although endoplasmic reticulum and Golgi apparatus were also implicated in early studies (Iwasa et al., 2001). Under extreme hypotonicity, other organelles including mitochondria and ER, but not lipid droplets, may also be vacuolated, suggesting the presence of constitutive or hypotonicity-induced water permeability in these compartments (King et al., 2020). However, under mild hypotonic conditions, vacuolation is restricted to lysosomes, suggesting that water influx to lysosomes selectively increases upon hypotonic stimulation (Li et al., 2020). Using Lucifer Yellow dextran dye that is selectively sensitive to heavy water (deuterium oxide; D_2_O), but not H_2_O, it was demonstrated that hypotonicity-induced vacuoles indeed uptake excess water from the cytosol (Li et al., 2020). When isolated native lysosomes are placed in water (i.e., extreme hypotonicity), lysosomes do burst, suggesting that increasing lysosomal water flux may cause lysosomal membrane rupture (Li et al., 2020). Importantly, Lyso-VRAC-deficient lysosomes are more resistant to hypotonic challenge (Li et al., 2020). Hence, lysosomes are intrinsically osmo-sensitive with regulated water permeability.

Both membrane fusion and osmotic swelling of lysosomes may contribute to the formation of hypotonicity-induced large vacuoles. Initial vacuoles must be osmotically swollen. However, the high membrane tension in the swollen lysosomes may promote membrane fusion (Chen et al., 2020; Li et al., 2020; Saric and Freeman, 2020). As membrane fusion may promote water influx, a positive feedback loop between membrane fusion and water influx may be formed (Li et al., 2020). Upon hypotonic challenge, lysosomes appear to “absorb” more water into their lumen, resulting in apparent increases of lysosome size (Li et al., 2020). Compared with other intracellular compartments, the lumen of lysosomes, rather than being water-based, might be partially matrix-based and osmosis-resistant, as observed in synaptic vesicles (Reigada et al., 2003; Taupenot et al., 2003).

Lyso-VRAC plays a key role in regulating lysosomal water flux, as hypotonicity-induced vacuolation was blocked when Lyso-VRACs are pharmacologically inhibited or genetically inactivated (Li et al., 2020). Baf-A1, a V-ATPase inhibitor that dissipates the lysosomal pH gradient, also prevented hypotonicity-induced vacuolation (Li et al., 2020), suggesting that H^+^-coupled solute transport may play a role. How does the channel activity of Lyso-VRAC facilitate water influx? Lysosomal Cl^−^ flux is likely important for hypotonicity-induced lysosomal vacuolation, yet the direction of Cl^−^ flux across lysosomal membranes is dependent on the electrochemical gradient of Cl^−^ in individual lysosomes (Li et al., 2020). It is also not clear whether Cl^−^-mediated changes in lysosomal Δψ might play a role. Lyso-VRACs are also permeable to other osmolytes, influx of which may also contribute to solute-coupled water influx. It is not known whether osmotic-obligation is the sole-coupling mechanism between lysosomal Cl^−^/osmolytes flux and water flux. It is worth noting that Lyso-VRAC itself may mediate a certain degree of water permeability (Nilius, 2004). It is also not clear whether anion flux may drive water uphill transport through Lyso-VRAC (Zeuthen, 2010), and whether Lyso-VRAC-mediated water permeability/conductance exhibits any rectification properties as the ionic conductance. In addition, the Lyso-VRAC is also permeable to bicarbonate (Li et al., 2020) that may play an important role in the regulation of water flux, because the influx of bicarbonate to the acidic lumen may result in a net influx of water (HCO_3_^−^ + H^+^ → H_2_O + CO_2_). Hence, hypotonicity-induced lysosomal vacuolation may occur as a result of lysosomal fusion and Lyso-VRAC-dependent anion and water flux across lysosomal membranes.

### Lysosomes in regulatory cytosolic volume decrease

Water storage capacity may allow lysosomes to play a uniquely important role in regulatory cytoplasmic volume decrease. The cytoplasm consists of the cytosol and intracellular compartments/organelles (see text box). We argue that as biochemical reactions require a normal volume constancy of cytoplasm, under the context of cell volume regulation, cytosolic volume is most relevant (Hoffmann et al., 2009; Jentsch, 2016). Cytosolic RVD can be accomplished by adjusting either the whole-cell volume, i.e., cytoplasmic volume, or the volume of intracellular organelles. Under hypotonic conditions, the contribution of organelles to RVD could be substantial. Because the extent of volume increases more than that of surface area, fusion of lysosomes can quickly increase the volume-to-surface-area ratio and scale up luminal water storage capacity (Li et al., 2020), effectively reducing the cytosolic volume after water inflowing to the lumen. Because Lyso-VRAC is required for hypotonicity-induced vacuolation and RVD, together with consistent blockade of hypotonicity-induced lysosomal vacuolation and RVD by multiple lysosome-acting reagents (Li et al., 2020), lysosomes are osmo-responsive organelles that contribute to RVD through vacuolation (Fig. 5). With repeated membrane fusion and osmotic swelling, watery lysosomes may serve as storage for excess intracellular water. The specificity of lysosomes acting as an excess water storage reservoir may be due to the high fusogenic potential of lysosomes (Luzio et al., 2007b), as well as its still hypothetic, quasi-matrix-based lumen (Reigada et al., 2003; Taupenot et al., 2003). Under hypotonic challenge, intracellular water may enter various organelles to cause their swelling. However, the feedforward regulation of water influx and membrane fusion in lysosomes may rapidly increase intracellular water storage. Consistently, fast fusion of small lysosomes to large vacuoles is observed under hypotonic stress (Li et al., 2020). Notably, large vacuoles can store much more water compared with a series of individual small lysosomes with equal membrane area (Fig. 4). However, these watery lysosomes are likely hypo-functional due to H^+^/hydrolase dilution, and the occupancy of a large portion of the cytoplasm. Therefore, although lysosomes may contribute to cytosolic RVD, hypotonicity-induced vacuolation is a temporary stress response.

### Active, capacitive water secretion in physiology and stress

Normal lysosomes can undergo lysosomal exocytosis, which is required for other cellular processes such as phagocytosis and membrane repair (Cheng et al., 2014; Samie et al., 2013; Yu et al., 2020). Watery vacuolated lysosomes may also expel water to the extracellular space through lysosomal exocytosis (Fig. 5). Water extrusion can occur passively through the plasma membrane water channels following the efflux of osmolytes. However, exocytosis of water-filled vacuolated lysosomes would not only reduce the cytoplasm volume but also provide intracellular membranes to reduce cell membrane stress. Hence, upon exocytosis of water-filled vacuolated lysosomes, lysosomes could also contribute to cytoplasmic RVD. Depending on the severity and duration of the insult, we suggest that cells may utilize both “passive” (mediated by plasma membrane water channels) and “active” (mediated by lysosomal water channels and exocytosis) mechanisms for water extrusion and cell survival.

Unlike apoptosis, which is regulated cell death accompanied by cell shrinkage, necrosis is accompanied by cell swelling (Galluzzi et al., 2018; Shubin et al., 2016). Organelle vacuolation, so-called cellular “edema,” is frequently observed in the necrotic cells (Galluzzi et al., 2018; Li et al., 2020; Shubin et al., 2016). Upon necrotic insults, such as thermal and hypoxic stress, Lyso-VRAC orchestrates pH-dependent lysosomal vacuolation, water sequestration, and exocytosis in order to prevent plasma membrane rupture (Li et al., 2020). When lysosome function is compromised, necrosis is dramatically increased (Li et al., 2020). On the other hand, boosting lysosome biogenesis might confer a resistance to necrosis. Hence, equipped with osmo-sensitive channels on the limited membranes, lysosomes could sequester and then extrude toxic levels of intracellular water, providing a regulatory mechanism that protects mammalian cells from necrotic injuries (Li et al., 2020). The exocytosis of hypotonicity-induced vacuoles may provide not only an active water extrusion mechanism to reduce osmotic stress but also a mechanism to reduce cell membrane tension by supplementing membranes (Groulx et al., 2006). Hence, if lysosomes mediate an active water extrusion mechanism and relief of membrane tension, lysosomal solute and water transport may play a more prominent role in cell survival than in osmo-regulation. In LSD cells, lysosomal vacuolation could be mildly activated as the cell’s adaptive response to genetic and environmental challenges (Xu and Ren, 2015). The identification of lysosomal solute/water transport mechanisms may provide a molecular foundation upon which to explore the links between lysosomal physiology, lysosome and cell volume regulation, intracellular water regulation, necrosis, and disease pathologies.

### Future directions

Molecular mechanisms that regulate ion flux in the lysosomes are beginning to be revealed, as well as their roles in regulating membrane fusion/fission and lysosomal swelling/condensation. We expect to see progress in the following areas: (1) molecular identities and regulation of lysosomal water channels (Lyso-AQPs) and catabolite transporters; (2) an assay to monitor lysosomal water flux during membrane fusion/fission and degradation; (3) the relationship between ionic composition/release and fusion/fission machinery, and between the osmotic regulation vs. membrane trafficking of lysosomes; (4) if lysosome-mediated water secretion plays a role in necrotic cell death, does lysosome enhancement have a general cell-protective role against necrotic insults?
